# 3,3′-Bis(4-fluoro­benz­yl)-1,1′-ethyl­enediimidazolium tribromidocuprate(I)

**DOI:** 10.1107/S1600536808021521

**Published:** 2008-07-16

**Authors:** Hon Man Lee, Chi-Ying Lu, Pi-Yun Cheng

**Affiliations:** a National Changhua University of Education, Department of Chemistry, Changhua 50058, Taiwan

## Abstract

The title compound, (C_22_H_22_F_2_N_4_)[CuBr_3_], crystallizes with the cation situated on an inversion center and the anion on a twofold rotation axis along one Cu—Br bond. The two imidazole rings are in an *anti* configuration. The anion has a trigonal planar coordination geometry.

## Related literature

For general background, see: Liao *et al.* (2007 ▶). For the structure of another salt of this cation, see: Lee *et al.* (2007 ▶).
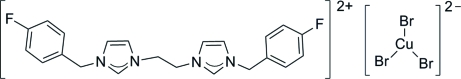


## Experimental

### 

#### Crystal data

(C_22_H_22_F_2_N_4_)[CuBr_3_]
*M* *_r_* = 683.71
Monoclinic, 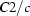

*a* = 15.4994 (11) Å
*b* = 11.0825 (8) Å
*c* = 15.4464 (12) Å
β = 117.582 (4)°
*V* = 2351.7 (3) Å^3^
*Z* = 4
Mo *K*α radiation
μ = 6.06 mm^−1^
*T* = 150 (2) K
0.30 × 0.21 × 0.19 mm

#### Data collection

Bruker SMART 1000 diffractometer
Absorption correction: multi-scan (*SADABS*; Sheldrick, 2001 ▶) *T*_min_ = 0.213, *T*_max_ = 0.318
11647 measured reflections
2686 independent reflections
2242 reflections with *I* > 2σ(*I*)
*R*_int_ = 0.023

#### Refinement

*R*[*F* ^2^ > 2σ(*F* ^2^)] = 0.032
*wR*(*F* ^2^) = 0.091
*S* = 1.03
2686 reflections
146 parameters
H-atom parameters constrained
Δρ_max_ = 1.51 e Å^−3^
Δρ_min_ = −0.73 e Å^−3^

### 

Data collection: *SMART* (Bruker, 2001 ▶); cell refinement: *SAINT* (Bruker, 2001 ▶); data reduction: *SAINT*; program(s) used to solve structure: *SHELXTL* (Sheldrick, 2008 ▶); program(s) used to refine structure: *SHELXTL*; molecular graphics: *SHELXTL*; software used to prepare material for publication: *SHELXTL*.

## Supplementary Material

Crystal structure: contains datablocks I, global. DOI: 10.1107/S1600536808021521/cf2209sup1.cif

Structure factors: contains datablocks I. DOI: 10.1107/S1600536808021521/cf2209Isup2.hkl

Additional supplementary materials:  crystallographic information; 3D view; checkCIF report

## Figures and Tables

**Table:** 

Cu1—Br2	2.3314 (9)
Cu1—Br1	2.3869 (5)

**Table:** 

Br2—Cu1—Br1	123.349 (16)
Br1—Cu1—Br1^i^	113.30 (3)

Symmetry code: (i) 
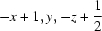
.

**Fig. 1.**
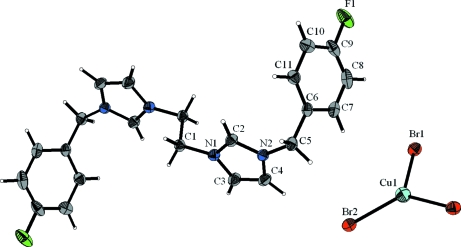
The structure of (I), showing 50% probability displacement ellipsoids for non-H atoms. H atoms are of arbitrary size. Unlabeled atoms of the imidazolium cation are related to labeled atoms by 3/2-x, 3/2-y, -z; for the anion, the symmetry operation for Br1 is 1-x + 1, y, 1/2-z.

## supplementary crystallographic information

### Comment

Our group is interested in the preparation of imidazolium salts, which can be employed as ligand precursors for *N*-heterocyclic carbenes (Liao *et al.*, 2007). In an unsuccessful attempt to prepare a copper carbene complex using 1,1'-bis(4-fluorobenzyl)-3,3'-ethylenediimidazolium dibromide, we isolated the title compound (I). Here we present its structure (Fig. 1). In our previous work (Lee *et al.*, 2007), we reported the structure of 1,1'-bis(4-fluorobenzyl)-3,3'-ethylenediimidazolium dichloride monohydrate (II), which features the same imidazolium cation with chloride anions.

Compound (I) crystallizes in the monoclinic space group *C*2/*c* with the imidazolium cation situated on a center of inversion. An notable feature is the anti configuration of the two imdazole rings, which is in contrast to the structure of (II). Also, there is no guest water incorporation as in the structure of (II). The anion lies on a twofold rotation axis and has trigonal-planar geometry.

### Experimental

The compound was prepared by heating a mixture of 1,1'-bis(4-fluorobenzyl)-3,3'-ethylenediimidazolium dibromide (0.10 g, 18.5 mmol), copper dibromide (0.0496 g, 22.2 mmol), and sodium acetate (0.0304 g, 37.0 mmol) in DMSO (5 ml) at 353 K for 4 h. After cooling, the solvent was removed completely under vacuum. The residual solid was washed with water and dichloromethane. Crystals were obtained by vapor diffusion of diethyl ether into a DMF solution of the compound.

### Refinement

All H atoms were positioned geometrically and refined with a riding model, with *U*_iso_(H) = 1.5*U*_eq_(C) for methyl H atoms and 1.2*U*_eq_(C) for all other H atoms.

### Crystal data

**Table:**

(C_22_H_22_F_2_N_4_)[CuBr_3_]	*F*_000_ = 1336
*M**_r_* = 683.71	*D*_x_ = 1.931 Mg m^−^^3^
Monoclinic, *C*2/*c*	Mo *K*α radiation λ = 0.71073 Å
Hall symbol: -C 2yc	Cell parameters from 3837 reflections
*a* = 15.4994 (11) Å	θ = 2.4–27.1º
*b* = 11.0825 (8) Å	µ = 6.06 mm^−^^1^
*c* = 15.4464 (12) Å	*T* = 150 (2) K
β = 117.582 (4)º	Prism, colorless
*V* = 2351.7 (3) Å^3^	0.30 × 0.21 × 0.19 mm
*Z* = 4	

### Data collection

**Table:**

Bruker SMART 1000 diffractometer	2686 independent reflections
Monochromator: graphite	2242 reflections with *I* > 2σ(*I*)
Detector resolution: 8.3 pixels mm^-1^	*R*_int_ = 0.023
*T* = 150(2) K	θ_max_ = 27.5º
ω scans	θ_min_ = 2.4º
Absorption correction: multi-scan(SADABS; Sheldrick, 2001)	*h* = −20→20
*T*_min_ = 0.214, *T*_max_ = 0.318	*k* = −14→14
11647 measured reflections	*l* = −20→20

### Refinement

**Table:**

Refinement on *F*^2^	Secondary atom site location: difference Fourier map
Least-squares matrix: full	Hydrogen site location: inferred from neighbouring sites
*R*[*F*^2^ > 2σ(*F*^2^)] = 0.032	H-atom parameters constrained
*wR*(*F*^2^) = 0.091	*w* = 1/[σ^2^(*F*_o_^2^) + (0.0522*P*)^2^ + 7.4402*P*] where *P* = (*F*_o_^2^ + 2*F*_c_^2^)/3
*S* = 1.03	(Δ/σ)_max_ < 0.001
2686 reflections	Δρ_max_ = 1.51 e Å^−^^3^
146 parameters	Δρ_min_ = −0.73 e Å^−^^3^
Primary atom site location: structure-invariant direct methods	Extinction correction: none

### Special details

**Table:**

Geometry. All e.s.d.'s (except the e.s.d. in the dihedral angle between two l.s. planes) are estimated using the full covariance matrix. The cell e.s.d.'s are taken into account individually in the estimation of e.s.d.'s in distances, angles and torsion angles; correlations between e.s.d.'s in cell parameters are only used when they are defined by crystal symmetry. An approximate (isotropic) treatment of cell e.s.d.'s is used for estimating e.s.d.'s involving l.s. planes.
Refinement. Refinement of *F*^2^ against ALL reflections. The weighted *R*-factor *wR* and goodness of fit *S* are based on *F*^2^, conventional *R*-factors *R* are based on *F*, with *F* set to zero for negative *F*^2^. The threshold expression of *F*^2^ > σ(*F*^2^) is used only for calculating *R*-factors(gt) *etc*. and is not relevant to the choice of reflections for refinement. *R*-factors based on *F*^2^ are statistically about twice as large as those based on *F*, and *R*- factors based on ALL data will be even larger.

### Fractional atomic coordinates and isotropic or equivalent isotropic displacement parameters (Å^2^)

**Table:**

	*x*	*y*	*z*	*U*_iso_*/*U*_eq_	
Cu1	0.5000	0.18316 (6)	0.2500	0.03387 (17)	
Br1	0.48562 (2)	0.06476 (3)	0.11487 (2)	0.02810 (12)	
Br2	0.5000	0.39352 (5)	0.2500	0.03037 (14)	
F1	0.8176 (2)	0.0493 (2)	0.03890 (16)	0.0474 (6)	
N1	0.71155 (19)	0.6850 (2)	0.08805 (18)	0.0213 (6)	
N2	0.7085 (2)	0.5320 (2)	0.17325 (18)	0.0213 (5)	
C1	0.7483 (2)	0.7770 (3)	0.0448 (2)	0.0224 (6)	
H1A	0.8143	0.8030	0.0933	0.027*	
H1B	0.7050	0.8484	0.0254	0.027*	
C2	0.7660 (2)	0.6007 (3)	0.1510 (2)	0.0241 (7)	
H2A	0.8343	0.5911	0.1759	0.029*	
C3	0.6162 (2)	0.6686 (3)	0.0690 (3)	0.0286 (7)	
H3A	0.5618	0.7158	0.0264	0.034*	
C4	0.6148 (3)	0.5730 (3)	0.1223 (3)	0.0287 (7)	
H4A	0.5591	0.5401	0.1241	0.034*	
C5	0.7439 (3)	0.4259 (3)	0.2389 (2)	0.0254 (7)	
H5A	0.6941	0.4014	0.2586	0.030*	
H5B	0.8039	0.4478	0.2986	0.030*	
C6	0.7651 (2)	0.3216 (3)	0.1889 (2)	0.0229 (7)	
C7	0.6915 (3)	0.2417 (3)	0.1315 (2)	0.0290 (7)	
H7A	0.6286	0.2509	0.1268	0.035*	
C8	0.7084 (3)	0.1489 (3)	0.0809 (3)	0.0355 (9)	
H8A	0.6581	0.0945	0.0416	0.043*	
C9	0.8001 (3)	0.1388 (3)	0.0898 (2)	0.0325 (8)	
C10	0.8756 (3)	0.2142 (3)	0.1463 (3)	0.0324 (8)	
H10A	0.9386	0.2033	0.1513	0.039*	
C11	0.8573 (2)	0.3071 (3)	0.1961 (2)	0.0268 (7)	
H11A	0.9082	0.3610	0.2352	0.032*	

### Atomic displacement parameters (Å^2^)

**Table:**

	*U*^11^	*U*^22^	*U*^33^	*U*^12^	*U*^13^	*U*^23^
Cu1	0.0247 (3)	0.0338 (4)	0.0381 (3)	0.000	0.0102 (3)	0.000
Br1	0.02629 (19)	0.0309 (2)	0.02709 (18)	−0.00222 (13)	0.01230 (14)	−0.00081 (13)
Br2	0.0272 (2)	0.0304 (3)	0.0374 (3)	0.000	0.0182 (2)	0.000
F1	0.0816 (19)	0.0265 (12)	0.0329 (11)	0.0060 (11)	0.0254 (12)	−0.0055 (9)
N1	0.0236 (13)	0.0230 (14)	0.0183 (12)	−0.0005 (11)	0.0107 (10)	0.0001 (10)
N2	0.0242 (13)	0.0216 (13)	0.0204 (12)	0.0010 (11)	0.0123 (10)	0.0022 (10)
C1	0.0282 (16)	0.0202 (16)	0.0216 (14)	−0.0029 (13)	0.0138 (12)	0.0006 (12)
C2	0.0242 (16)	0.0266 (17)	0.0220 (15)	0.0003 (13)	0.0112 (12)	0.0039 (12)
C3	0.0228 (16)	0.0292 (18)	0.0350 (18)	0.0036 (14)	0.0144 (14)	0.0086 (14)
C4	0.0233 (16)	0.0311 (18)	0.0371 (18)	0.0013 (14)	0.0185 (14)	0.0046 (15)
C5	0.0322 (18)	0.0232 (17)	0.0238 (15)	0.0011 (14)	0.0156 (14)	0.0059 (13)
C6	0.0271 (16)	0.0210 (16)	0.0208 (14)	−0.0014 (13)	0.0112 (12)	0.0042 (12)
C7	0.0268 (17)	0.0274 (18)	0.0300 (17)	−0.0066 (14)	0.0107 (13)	0.0044 (14)
C8	0.044 (2)	0.0245 (18)	0.0269 (17)	−0.0103 (16)	0.0068 (15)	0.0037 (14)
C9	0.055 (2)	0.0176 (16)	0.0223 (15)	0.0036 (16)	0.0151 (15)	0.0021 (13)
C10	0.0349 (19)	0.031 (2)	0.0313 (17)	0.0040 (15)	0.0155 (15)	−0.0002 (15)
C11	0.0260 (17)	0.0248 (17)	0.0259 (15)	−0.0017 (13)	0.0089 (13)	−0.0024 (13)

### Geometric parameters (Å, °)

**Table:**

Cu1—Br2	2.3314 (9)	C3—H3A	0.950
Cu1—Br1	2.3869 (5)	C4—H4A	0.950
Cu1—Br1^i^	2.3869 (5)	C5—C6	1.508 (5)
F1—C9	1.368 (4)	C5—H5A	0.990
N1—C2	1.331 (4)	C5—H5B	0.990
N1—C3	1.379 (4)	C6—C7	1.390 (5)
N1—C1	1.470 (4)	C6—C11	1.391 (5)
N2—C2	1.333 (4)	C7—C8	1.388 (6)
N2—C4	1.370 (4)	C7—H7A	0.950
N2—C5	1.482 (4)	C8—C9	1.369 (6)
C1—C1^ii^	1.532 (6)	C8—H8A	0.950
C1—H1A	0.990	C9—C10	1.373 (5)
C1—H1B	0.990	C10—C11	1.390 (5)
C2—H2A	0.950	C10—H10A	0.950
C3—C4	1.349 (5)	C11—H11A	0.950
			
Br2—Cu1—Br1	123.349 (16)	N2—C5—C6	110.9 (3)
Br2—Cu1—Br1^i^	123.349 (16)	N2—C5—H5A	109.5
Br1—Cu1—Br1^i^	113.30 (3)	C6—C5—H5A	109.5
C2—N1—C3	108.6 (3)	N2—C5—H5B	109.5
C2—N1—C1	124.8 (3)	C6—C5—H5B	109.5
C3—N1—C1	126.6 (3)	H5A—C5—H5B	108.0
C2—N2—C4	108.7 (3)	C7—C6—C11	119.1 (3)
C2—N2—C5	123.3 (3)	C7—C6—C5	120.4 (3)
C4—N2—C5	127.9 (3)	C11—C6—C5	120.5 (3)
N1—C1—C1^ii^	108.8 (3)	C8—C7—C6	121.2 (3)
N1—C1—H1A	109.9	C8—C7—H7A	119.4
C1^ii^—C1—H1A	109.9	C6—C7—H7A	119.4
N1—C1—H1B	109.9	C9—C8—C7	117.5 (3)
C1^ii^—C1—H1B	109.9	C9—C8—H8A	121.2
H1A—C1—H1B	108.3	C7—C8—H8A	121.2
N1—C2—N2	108.4 (3)	F1—C9—C8	118.3 (3)
N1—C2—H2A	125.8	F1—C9—C10	118.0 (4)
N2—C2—H2A	125.8	C8—C9—C10	123.6 (4)
C4—C3—N1	107.0 (3)	C9—C10—C11	118.0 (4)
C4—C3—H3A	126.5	C9—C10—H10A	121.0
N1—C3—H3A	126.5	C11—C10—H10A	121.0
C3—C4—N2	107.4 (3)	C10—C11—C6	120.5 (3)
C3—C4—H4A	126.3	C10—C11—H11A	119.8
N2—C4—H4A	126.3	C6—C11—H11A	119.8

Symmetry codes: (i) −*x*+1, *y*, −*z*+1/2; (ii) −*x*+3/2, −*y*+3/2, −*z*.
